# Using a volitional help sheet to increase university students' attendance at on‐campus lectures: A randomized controlled trial

**DOI:** 10.1111/bjep.70014

**Published:** 2025-08-01

**Authors:** Mark A. Elliott, Allan McGroarty, David J. Robertson, Hazel P. Anderson

**Affiliations:** ^1^ Department of Psychological Sciences and Health University of Strathclyde Glasgow UK

**Keywords:** encoding facilitation, implementation intentions, lecture attendance, memory, volitional help sheet

## Abstract

**Background:**

A volitional help sheet (VHS) is an intervention that promotes the formation of implementation intentions. Research has established that VHSs can change a range of behaviours, including increased attendance at online university lectures. However, research has not yet tested whether VHSs can increase attendance at on‐campus lectures. Additionally, no studies have explicitly tested the extent to which memory improvement techniques can increase the efficacy of VHSs, nor have they tested the dependence of VHSs on memory. However, models of memory dictate that rehearsal of specified implementation intentions should boost their efficacy and that their efficacy should depend on memory ability.

**Aims:**

To test whether: (1) a VHS can increase university students' attendance at on‐campus lectures; (2) the efficacy of the VHS could be boosted with an encoding facilitation task requiring rehearsal (articulatory and elaborative) of implementation intentions; and (3) memory ability moderates the effects of the VHS on lecture attendance.

**Materials and Methods:**

Students enrolled in an undergraduate Psychology degree programme (*N* = 252) completed online measures of goal intention to attend lectures and both self‐reported and objective memory ability. These students were subsequently randomised to receive a VHS only, a VHS plus encoding facilitation or a control intervention, designed to increase lecture attendance.

**Results:**

Both VHS conditions attended a greater proportion of lectures over the following 11‐week teaching semester than did the control condition. There was no difference in lecture attendance rates between the VHS only and VHS plus encoding facilitation conditions. Memory ability did not moderate the effects of the VHS on lecture attendance rates.

**Discussion and Conclusion:**

The VHS was efficacious at increasing on‐campus university lecture attendance. There was no evidence that the encoding facilitation task boosted the effects of the VHS or that the efficacy of the VHS was dependant on memory ability. VHSs are likely to constitute useful interventions for increasing university students' attendance at on campus lectures. Further research could usefully test the efficacy of memory improvement techniques and the dependency of VHSs in samples with memory difficulties, where there is likely to be more scope for improvement than in the university student populations. Further research is also required to test other ways to boost the efficacy of VHSs.

## INTRODUCTION

University lectures are associated with valued pedagogical objectives (e.g., Charlton, 2006; Webster, 2015), and as a result, academic performance (e.g., Credé et al., 2010; Edwards & Clinton, 2019; Louis et al., 2016; Romer, 1993; Tokumitsu, 2017; Trice et al., 2000; Wongtrakul & Dangprapai, 2020). However, attendance rates are typically found to be low, often around 50% (e.g., Beovich et al., 2021; Hollett et al., 2020; Skead et al., 2020; Williams, 2022) and as low as 36% (Moore et al., 2008). This research therefore aimed to test the efficacy of an intervention, namely, a volitional help sheet (VHS; Armitage, 2008) for increasing university lecture attendance rates.

### Volitional help sheets

A VHS (Armitage, 2008) is an intervention that converts goal intentions (e.g., “I intend to attend my lectures this semester”) into action through the promotion of implementation intentions (IF‐THEN plans; Gollwitzer, 1993). It provides a list of pre‐specified critical situations that put an intended behaviour (e.g., attending a lecture) at risk. These critical situations are presented as IF statements (e.g., ‘IF I am tempted to miss a lecture because I have a university deadline approaching’). It also contains a list of pre‐specified goal‐directed responses (strategies) that can be used to manage the critical situations. The goal‐directed responses are presented as THEN statements (e.g., THEN I will remind myself that attending lectures should help me get a higher mark). Recipients of a VHS are asked to form implementation intentions by selecting the IF statements that correspond to the critical situations that might put their performance of an intended behaviour at risk, and linking each one with a THEN statement. This creates mental representations of the specified critical situations and links to the specified goal‐directed responses. These mental representations are encoded *to* memory and subsequently activated *from* memory when people encounter the specified critical situations. This helps ensure goal‐intended behaviour by increasing the salience of the selected critical situations when they are encountered and automatically initiating the linked goal‐directed response (e.g., Gollwitzer & Sheeran, 2006; Webb & Sheeran, 2004). As a result, implementation intentions change behaviour by helping people to overcome self‐regulatory problems that stop them from enacting their goal intentions, such as missing opportunities to act or getting tempted by alternative courses of action (Sheeran & Webb, 2016).

VHSs have been found to successfully improve a range of behaviours (e.g., Arden & Armitage, 2012; Armitage, 2008; Armitage et al., 2016, 2017; Armitage & Arden, 2010; Brewster et al., 2015, 2016; Elliott et al., 2021; Paterson et al., 2023). However, just one previous study has tested whether VHSs can modify educational behaviours. Elliott et al. (2024) allocated 178 university students at random to either a VHS or control condition. The participants in the VHS condition were presented with a list of 20 critical situations (reasons for lecture absenteeism) that were derived from previous research evidence (e.g., Bati et al., 2013; Moore et al., 2008; Skead et al., 2020) and a list of 24 goal‐directed responses that were based on Prochaska and DiClemente's (1983) processes of behaviour change. They were asked to select four critical situations and pair each one with a goal‐directed response, thus forming four implementations to support lecture attendance. The participants allocated to the control condition were asked to select the same number of critical situations and goal‐directed responses from the VHS but without linking the two. In support of the VHS's efficacy, the participants in the VHS condition were found to attend a greater proportion of synchronous (live) online lectures over the preceding 11‐week teaching semester (59% attendance rate) than did the control condition (48% lecture attendance rate).

While Elliott et al.'s (2024) study provides encouraging evidence in support of the efficacy of VHSs in higher education for increasing lecture attendance rates, further evidence is required for three key reasons. First, Elliott et al. (2024) is the only previous study in the educational literature to have tested the efficacy of a VHS, so a replication of the results is necessary to inform evidence‐based practice (e.g., Bauer & Prenzel, 2012; Davies, 1999). Second, the study was not pre‐registered, meaning that a pre‐registered replication of the results would be beneficial for scientific integrity (Open Science Collaboration, 2015). Third, the study focused on attendance at online lectures, whereas on‐campus lectures remain the main mode of delivery in higher education (e.g., Goffe & Kauper, 2014; Watts & Schaur, 2011). While synchronous online lectures share many similarities to on‐campus lectures (e.g., they are delivered live, within a formal timetable), there are also differences that might impact the efficacy of VHSs. For example, attendance at on‐campus lectures requires travel and greater personal interaction with lecturers and other students. In addition, it is possible for students to log into online lectures but switch off their video camera, meaning that they could falsify their attendance. Therefore, there are potential economic, social, and volitional factors associated with on‐campus lectures that differentiate them from their online counterparts and make the promotion of lecture attendance a greater self‐regulatory challenge. The present, pre‐registered, study therefore sought to test whether the efficacy of Elliott et al.'s (2024) VHS could be extended to on‐campus lecture attendance.

### Encoding facilitation and memory ability

This study also tested whether increases in lecture attendance rates, following administration of the VHS, could be boosted with an encoding facilitation intervention. As stated above, VHSs ask people to form implementation intentions by making links between specified critical situations and goal‐directed responses. These links are encoded to memory and subsequently activated to help ensure the performance of a goal‐intended behaviour when the specified critical situations are encountered. Memory ability is therefore likely to play an important role in determining the success of a VHS. In particular, models of memory (e.g. Atkinson & Shiffrin, 1968; Baddeley et al., 2020) postulate that information (e.g., mental links between specified critical situations and goal‐directed responses) must be transferred from short‐term or working memory (e.g., during the initial specification of an implementation intention) to long‐term memory, where it is stored, enabling its later use (e.g., the activation of the mental links between the specified critical situations and goal‐directed responses when the specified critical situations are encountered).

Mental rehearsal is required to facilitate the successful encoding of information to long‐term memory (e.g. Atkinson & Shiffrin, 1968; Craik & Lockhart, 1972). Articulatory, or maintenance, rehearsal involves repeating information in its original form (e.g., re‐reading an implementation intention that has been specified with the assistance of a VHS). It is passive and reflects shallow processing, designed to enable information to be retained in short‐term or working memory until it is no longer needed (e.g., Baddeley, 2012; Logie, 2011). Elaborative rehearsal involves linking new information to information that has already been learned (e.g., re‐specifying, anew, an implementation intention that has been specified previously). It is active and reflects deep processing, which is necessary for transferring information to long‐term memory (e.g., Anderson & Reder, 1979; Craik & Lockhart, 1972). Evidence shows that articulatory rehearsal does not improve memory performance but elaborative rehearsal does (e.g., Craik & Tulving, 1975; Peterson & Peterson, 1959). However, while participants in some previous studies (e.g., O'Connor et al., 2017) have been asked to re‐read their stated implementation intentions (articulatory rehearsal) or re‐specify them anew, after initial specification (elaborative rehearsal), no previous studies have tested whether mental rehearsal boosts the efficacy of implementation intention only interventions (e.g., VHSs). In this study, we therefore tested, for the first time, whether an encoding facilitation task, encouraging both articulatory and elaborative rehearsal of implementation intentions, could boost the efficacy of Elliott et al.'s (2024) VHS at increasing lecture attendance.

Also, given the theoretical importance of memory ability in the successful encoding and activation of implementation intentions, it is likely that VHSs will constitute more effective behaviour change interventions for people with better (versus poorer) memory ability. Previous research on implementation intentions has given little empirical attention to this issue. While the effect of implementation intention specification on enhanced memory performance has been tested previously (e.g., Burkard et al., 2014; Meeks et al., 2015), the dependence of implementation intention specification, as a behaviour change strategy, on memory has been tested, to date, in just one previous study. Hall et al. (2014) found that participants who formed implementation intentions to increase their physical activity and who had high levels of executive function (e.g., working memory ability) engaged subsequently in a greater amount of physical activity, compared with participants who had low levels of executive function. Consistent with this one study from the domain of physical activity, we tested the hypothesis that memory ability will increase the extent to which Elliott et al.'s (2024) VHS generates improvements in on‐campus lecture attendance.

### Hypotheses

Consistent with the above review, and as pre‐registered on the Open Science Framework (OSF: https://osf.io/t6s4y), the hypotheses were:

Hypothesis 1 *Participants randomized to a VHS only condition, and asked to specify implementation intentions, will subsequently attend a greater proportion of lectures than will participants randomized a control condition (i.e., the VHS will increase on campus lecture attendance rates)*.

Hypothesis 2 *Participants randomized to a VHS plus encoding facilitation condition, requiring both articulatory and elaborative rehearsal of their specified implementation intentions, will subsequently attend a greater proportion of lectures than will participants randomized to both the VHS only and control conditions (i.e., the encoding facilitation intervention will boost the effectiveness of the VHS at increasing lecture attendance rates)*.

Hypothesis 3 *Memory ability will moderate the effects of condition on the proportion of lectures attended: higher levels of memory ability will be associated with greater differences in lecture attendance between the VHS only and control conditions (i.e., memory ability will increase the extent to which the VHS generates improvements in lecture attendance; note that memory ability was not expected to moderate the differences between the VHS plus encoding facilitation condition and the other conditions because the encoding facilitation task should, theoretically, resolve any problems caused by poor memory ability in the encoding of implementation intentions)*.

## METHOD

The method was in accordance with the pre‐registration on the Open Science Framework.

### Participants

**FIGURE 1:**
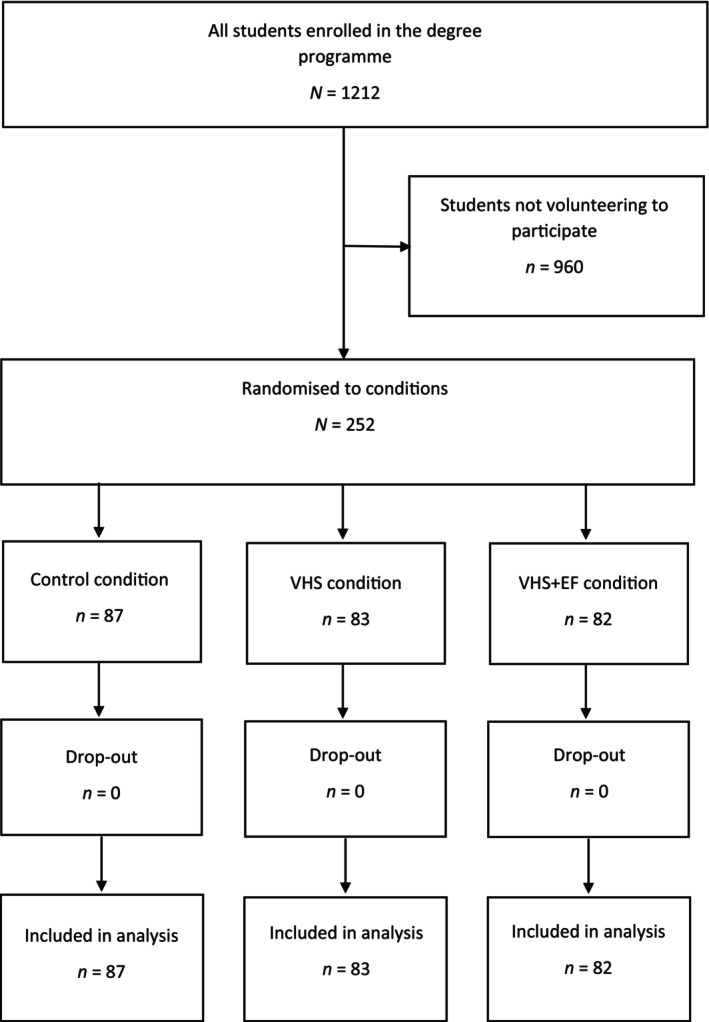
CONSORT flow chart.

The sample comprised *N* = 252 university students who were enrolled in a 4‐year undergraduate Psychology degree programme at a large University in Scotland (see Figure 1 for the CONSORT flow chart). Table 1 shows the profile of the sample and the population (i.e., all students on the degree programme) in terms of age, gender, and year of study. As the table shows, the profile of the sample closely matched the profile of the population.^1^

**TABLE 1:** Sample and population characteristics.

		Sample (*N* = 252)	Population (*N* = 1212)
Age	Mean (SD)	19.96 (4.57)	20.21 (3.75)
Gender			
Males	*n* (%)	32 (13)	237 (20)
Females	*n* (%)	218 (87)	970 (80)
Other	*n* (%)	2 (1)	n/a
Year of study			
1st year	*n* (%)	96 (38)	513 (42)
2nd year	*n* (%)	85 (34)	306 (25)
3rd year	*n* (%)	47 (19)	253 (21)
4th year	*n* (%)	24 (10)	140 (12)

*Note*: University records provided data on sex (i.e., male or female) only, hence the “n/a” entry for the “other” option in the population's gender profile.

### Design and procedure

A randomized controlled design was used. The participants were sampled from all four levels of the undergraduate Psychology programme. The programme comprised two 11‐week teaching semesters per academic year. Semester 1 ran from mid‐September to early December. Semester 2 ran from mid‐January to late March. Semester 1 was preceded by a ‘welcome and development week’ and semester 2 by a ‘consolidation and development week’. For students at levels 3 and 4 of the programme, advertisements were posted within the virtual learning environments of the students' classes during the consolidation and development week of semester 2, in the 2023–2024 academic year. For students at levels 1 and 2 of the programme, the same sampling procedure was used but during the welcome and development week of semester 1, in the 2024–2025 academic year.^2^ The advertisements contained a link to an online questionnaire.

The online questionnaire took 5 min to complete and was designed and administered using Qualtrics. At the start of the questionnaire, the participants were presented with an information sheet, followed by a consent form. The information sheet explained that the study was an investigation into how it might be possible to increase students' lecture attendance and provided standard information on ethical rights and data protection. The participants who consented to proceed with the study were asked for their first and last names to enable their questionnaire data to be matched with data from other sources about lecture attendance, age, gender, and level of study (see below).

Next, the participants were presented with items to measure goal intentions to attend lectures over the course of the proceeding academic semester and self‐reported memory ability. They were then presented with an objective test of memory. After completing these items, they were allocated at random, by the survey software, to one of three conditions: a VHS condition; a VHS plus encoding facilitation condition; or a control condition.

The participants allocated to the VHS condition (*n* = 83) were asked to complete Elliott et al.'s (2024) VHS. They were presented with a series of drop‐down menus, containing 20 critical situations that can tempt students to miss a lecture and 24 goal‐directed responses that can be employed to overcome the temptation. These were presented as first‐person IF statements (e.g., IF I am tempted to miss a lecture because I have a university deadline approaching) and THEN statements (e.g., THEN I will remind myself that attending lectures should help me get a higher mark), respectively. The participants were asked to specify four implementation intentions by selecting four critical situations and linking each one with a goal‐directed response (for a full description of the VHS see Elliott et al., 2024).

The participants allocated to the VHS plus encoding facilitation condition (*n* = 82) were also asked to complete Elliott et al.'s (2024) VHS. In addition, after specifying each implementation intention, they were asked to read it three times, thus promoting articulatory rehearsal (Baddeley, 2012; Logie, 2011). To promote elaborative rehearsal (Anderson & Reder, 1979; Craik & Lockhart, 1972), they were then told to click on a NEXT button, which would take them to a new screen, on which they would be asked to restate the critical situation and goal‐directed response that they had just selected. When they clicked on the NEXT button, they were presented with the full list of 20 critical situations and 24 goal‐directed responses in the VHS. They were then asked to confirm which critical situation and goal‐directed response they had just linked together.

The participants allocated to the control condition (*n* = 87) were also presented with the same critical situations and goal‐directed responses contained with Elliott et al.'s (2024) VHS. However, to help ensure these participants did not form implementation intentions, the critical situations and goal‐directed responses were not presented as first‐person IF and THEN statements. The critical situations were presented as third‐person WHEN statements (e.g., most students would be tempted to miss a lecture WHEN they have a university deadline approaching). The goal‐directed responses were presented as third‐person strategy statements (e.g., to help them avoid missing lectures, most students would find it effective to ‘remind themself that attending lectures should help them get a higher mark’). Also, the control participants were not asked to link critical situations with goal‐directed responses. Instead, they were asked to select four critical situations, and separately, four goal‐directed responses.

Lecture attendance was recorded at each lecture over the 11 week teaching semester following either the welcome and development week (for levels 1 and 2 of the programme in the 2024–25 academic year) or the consolidation and development week (for levels 3 and 4 of the programme in the 2023–24 academic year). At the end of the 11 week teaching semester, the data from the online questionnaires were downloaded from Qualtrics into SPSS, and merged with the lecture attendance data. They were also merged with information from official university records about age, gender, and year of study. Finally, the participants were debriefed and the data were anonymized.

### Measures

#### Goal intentions

Consistent with previous research on implementation intentions and Elliott et al.'s (2024) previous research on lecture attendance, goal intentions were measured with standard items in line with Fishbein and Ajzen's (2010) recommendations. The participants responded to four items (e.g., ‘How many lectures do you intend to go to this semester?’) using a 9‐point unipolar scale (e.g., 1 = none of them to 9 = all of them). The mean of the four items was used as the final measure of goal intention (*α* = .62).

#### Memory ability

To increase the validity of the findings, memory ability was measured using both self‐reports and an objective test. Self‐report memory ability was measured with the prospective and retrospective memory questionnaire (PRMQ; Smith et al., 2000). This is an established measure of memory ability (e.g., Piauilino et al., 2010). It taps into people's ability to remember to perform an action in the future (prospective memory) and their ability to remember past events (retrospective memory). The participants were told: “These questions are about memory mistakes that people can make. Please indicate how often these kinds of things happen to you”. They were then presented with the eight items to measure prospective memory mistakes (e.g., ‘How often do you decide to do something in a few minutes’ time and then forget to do it?) and eight items to measure retrospective memory mistakes (e.g., ‘How often do you fail to recognise a place you have visited before?’). The participants responded to all items using 5‐point Likert scales with 1 = very often, 2 = quite often, 3 = sometimes, 4 = rarely, and 5 = never. Higher scores therefore corresponded to fewer errors in memory (i.e., better memory ability). The mean of the eight prospective and eight retrospective memory items was used as the final measure of self‐reported memory ability (*α* = .90).

Objective memory ability was measured using an established, short, non‐word encoding and recognition memory test (see Angwin et al., 2017). During the learning phase of the test, the participants were asked to learn a block of 24 non‐words (e.g., huly). The online questionnaire was programmed so that each non‐word was presented, in turn, on the screen, for 5 seconds, with a blank screen between each non‐word. The participants were told that they needed to attend carefully to each non‐word and that their recognition would be tested subsequently. During the recognition phase of the test, the participants were presented with the 24 target (learned) items and 24 foils, one at a time, in a random sequence. They were asked to indicate if the presented non‐word had been learned previously in the learning phase. This permitted an objective measure of individual differences in encoding proficiency using a neutral item set that did not place demands on existing language proficiency or declarative knowledge. The overall proportion of correct responses (i.e., acceptance of the target items and rejection of the foils) was used as the final measure of objective memory ability.

#### Lecture attendance

Lecture attendance was measured using the University's attendance monitoring system. The University's attendance monitoring system requires a QR code to be presented on the projector screen during each lecture. Students scan the QR code using their mobile phones. The QR codes take them to their personal account in the attendance monitoring system where they indicate their lecture attendance. Each lecture has a unique QR code, which changes every 5 seconds,^3^ meaning that students can register only their attendance at the lecture that is being delivered. The final measure of lecture attendance was the proportion of lectures that the participants attended over the course of the relevant 11‐week teaching semester.

## RESULTS

The data were analysed in accordance with the pre‐registration on the Open Science Framework.

### Tests of randomisation

A series of one‐way ANOVAs and Chi‐squared tests determined whether there were any differences between the conditions prior to intervention. The dependent variables in the ANOVAs were: goal intention (ANOVA 1), self‐reported memory ability (ANOVA 2), objective memory ability (ANOVA 3), and age (ANOVA 4). The independent variable in each analysis was condition (0 = active control; 1 = VHS; 2 = VHS plus encoding facilitation). The variables in the Chi‐squared tests were condition by gender (test 1) and condition by academic year of study (test 2). There were no significant differences between the conditions on any measure (see Table 2). Randomization to the conditions was therefore successful.

**TABLE 2:** Descriptive statistics and tests of difference between the conditions.

		CONT *n* = 87	VHS *n* = 83	VHS + EF *n* = 82	Tests of difference
Proportion of lectures attended	Mean (SD):	63.53 (32.49)†	76.13 (23.45) ±	74.30 (25.31) ±	*F* (df = 6, 245) = 6.62, MSE = 619.02, *p* = .002, *Cohen's f* = .21^a^
Goal intention (scale range: 1–9)	Mean (SD)	8.30 (.86)	8.36 (.96)	8.41 (.86)	*F* (df = 2, 249) = .36, MSE = .80, *p* = .701, *Cohen's f* = .05^b^
Self‐reported memory ability (scale range: 1–5)	Mean (SD)	3.18 (.70)	3.12 (.62)	3.09 (.66)	*F* (df = 2, 249) = .42, MSE = .44, *p* = .656, *Cohen's f* = .08^b^
Objective memory ability (% targets and foils identified correctly)	Mean (SD)	80.07 (9.04)	81.78 (9.06)	81.83 (10.04)	*F* (df = 2, 249) = .96, MSE = 88.13, *p* = .386, *Cohen's f* = .09^b^
Age	Mean (SD)	19.77 (3.89)	19.92 (4.66)	20.21 (5.14)	*F* (df = 2, 249) = .20, MSE = 20.98, *p* = .820, *Cohen's f* = .04^b^
Gender					
Males	*n* (%)	9 (10)	15 (18)	8 (10)	χ^2^ (df = 4) = 6.96, *p* = .138, *V* = .12^c^
Females	*n* (%)	76 (87)	68 (82)	74 (90)	
Other	*n* (%)	2 (2)	0 (0)	0 (0)	
Year of study					
1st year	*n* (%)	31 (36)	30 (36)	35 (43)	χ^2^ (df = 6) = 2.06, *p* = .914, *V* = .06^c^
2nd year	*n* (%)	32 (37)	27 (33)	26 (32)	
3rd year	*n* (%)	16 (18)	16 (19)	15 (18)	
4th year	*n* (%)	8 (9)	10 (12)	6 (7)	

*Note*: †, ± = means with different symbols differ significantly at *p* < .01. *Cohen's f* = .10, .25 and .4 are the criteria for small‐, moderate‐ and large‐sized effects in the social sciences, respectively (Cohen, 1992).

Abbreviations: CONT, active control condition; EF, encoding facilitation task; VHS, volitional help sheet condition.

^a^ ANCOVA with the correlates of lecture attendance (goal intention, prospective memory ability, retrospective memory ability and year of study) as covariates.

^b^ ANOVAs.

^c^ Chi‐squared tests.

### Descriptive statistics

The participants in each condition had extremely positive goal intentions towards attending lectures (means towards the top end of the scale), moderate levels of self‐reported memory ability (means just above the middle of the scale), and high levels of performance (>80% accurate recall) on the objective memory test (see Table 1). On average, 64% of the lectures were attended by the participants in the control condition, 76% were attended by the participants in the VHS only condition, and 74% were attended by the participants in the VHS plus encoding facilitation condition.

### Effects of the VHS on lecture attendance

Hypotheses 1 and 2 were tested simultaneously with a one‐way ANCOVA. The dependent variable was the measure of lecture attendance. The independent variable was condition (0 = control; 1 = VHS only; 2 = VHS plus encoding facilitation). The measures of goal intention, self‐report memory ability, and year of study were included as covariates because they were correlated significantly with the dependent variable (see Table 3). As shown in Table 2, there was a statistically significant difference between the conditions. In support of Hypothesis 1, post‐hoc comparisons showed that the VHS only condition attended a greater proportion of lectures (adjusted *M* = 76.23, SE = 2.74) than did the control condition (adjusted *M* = 63.38, SE = 2.68; *p* for difference = .001). However, while the VHS plus encoding facilitation condition also attended a greater proportion of lectures than did the control condition (adjusted *M* = 74.39, SE = 2.77; *p* for difference = .008), Hypothesis 2 was not supported because there was no difference between the two VHS conditions (*p* for difference = .459).

**TABLE 3:** Correlation matrix.

Variable	*r* (*p*)					
	1	2	3	4	5	6
1. Proportion of lectures attended	–	.32 (<.001)	.25 (.001)	.15 (.057)	−.11 (.138)	−.14 (.069)
2. Goal intention		–	.12 (.113)	.09 (.227)	.00 (.998)	−.01 (.895)
3. Self‐reported memory ability			–	.15 (.056)	−.03 (.712)	.11 (.160)
4. Objective memory ability				–	.02 (.841)	.17 (.168)
5. Age					–	.09 (.229)
6. Year of Study						–

### Moderation by memory ability

Hypothesis 3 was tested with a moderated linear regression (see Table 4). The dependent variable was the measure of lecture attendance. The independent variables were condition (0 = control, 1 = VHS only), the measures of self‐reported and objective memory ability, and the two‐way interactions between condition, on the one hand, and each of the two memory ability measures, on the other. Year of study was also included as an independent variable, given its significant association with lecture attendance (see Table 3). Consistent with standard procedures to reduce multicollinearity (Aiken & West, 1991), the continuous (memory ability) measures were mean centred prior to calculating the two‐way interactions.

**TABLE 4:** Moderated linear regression predicting proportion of lectures attended from condition, self‐reported and objective memory ability and the interactions between condition and each memory measure.

Variable	*R* ^2^	*F* (df = 6, 163)	*p* for *F*	*β*	*t*	*p* for *t*
Condition (0 = CONT, 1 = VHS)	.16	5.20	<.001	.23	3.19	.002
Year of study				−.16	−2.23	.027
Self‐reported memory ability				.31	3.19	.002
Objective memory ability				.01	.11	.915
Condition × self‐reported memory ability				−.04	−.41	.682
Condition × objectively measured memory				.08	.79	.433

Abbreviations: CONT, active control condition; VHS, volitional help sheet only condition.

As Table 4 presents, the independent variables accounted for a significant percent of the variance in the measure of lecture attendance. Consistent with the ANCOVA reported above, condition was a significant predictor; the standardized regression weight showed that the VHS condition attended a greater proportion of lectures than did the control condition (see Table 4). Year of study and self‐reported memory were also significant independent predictors; objective memory ability was not. The standardized regression weights for year of study and self‐reported memory ability indicated that lecture attendance decreased with year of study and increased with self‐reported memory ability. Contrary to Hypothesis 3, the two‐way interactions between condition and self‐reported memory ability and condition and objective memory ability were not statistically significant.

## DISCUSSION

In this study, we tested whether a VHS, designed to promote the formation of implementation intentions, could increase university students' attendance at on‐campus lectures. We also tested whether an encoding facilitation intervention, which required participants to rehearse and re‐specify the implementation intentions that they had formed using the VHS, could further increase lecture attendance rates, and whether memory ability moderated the effectiveness of the VHS on lecture attendance rates. It was hypothesized that participants randomized to a VHS only condition would subsequently attend a greater proportion of lectures than would participants randomized to a control condition (Hypothesis 1). It was also hypothesized that participants randomized to a VHS plus encoding facilitation condition would subsequently attend a greater proportion of lectures than would participants randomized to both the VHS and control conditions (Hypothesis 2). Finally, it was hypothesized that memory ability would moderate the effects of condition on the proportion of lectures attended, with higher levels of memory ability being associated with a higher attendance rate in the VHS only condition, relative to the control condition (Hypothesis 3).

In support of Hypothesis 1, university students who were allocated at random to a VHS only condition attended a significantly greater proportion of lectures over the subsequent 11‐week teaching semester than did university students who were allocated at random to a control condition (76% versus 63%). These findings are consistent with previous studies, which have shown that VHSs constitute useful interventions for changing behaviour in other, non‐educational contexts (e.g., Arden & Armitage, 2012; Armitage, 2008; Armitage et al., 2016, 2017; Armitage & Arden, 2010; Brewster et al., 2015, 2016; Elliott et al., 2021; Paterson et al., 2023). They are also consistent with the findings of Elliott et al. (2024), who showed that the current VHS can increase university students' attendance at online lectures. However, this study extends Elliott et al.'s (2024) findings by showing that the VHS can increase lecture attendance in a setting (i.e., on‐campus) that is associated with economic (e.g., travel costs), volitional (e.g., effort) and social (e.g., interactions with students and lecturers) factors that can deter lecture attendance, and therefore, make behavioural performance (i.e., lecture attendance) more challenging. Nevertheless, in spite of these additional challenges, the VHS was still found to generate a 13% point increase in attendance rates. The present study therefore provides strong evidence for the efficacy of the VHS with regards to supporting university students' lecture attendance.

Hypotheses 2 and 3 were not supported. First, university students who were allocated at random to the VHS plus encoding facilitation condition attended a greater proportion of lectures than did university students who were allocated at random to the control condition. However, there was no evidence that their lecture attendance rates differed, on average, from university students who were allocated at random to the VHS only condition. These findings therefore provide further evidence in support of the efficacy of the VHS at increasing lecture attendance rates, but they do not provide evidence that the encoding facilitation intervention boosted its efficacy. Second, neither self‐reported nor objective measures of memory ability moderated the effects of condition (VHS only versus control) on lecture attendance rates. Contrary to expectations, therefore, there was no evidence that better memory ability resulted in greater efficacy of the VHS for increasing lecture attendance rates.

While the null results might imply that memory ability is unimportant in dictating the efficacy of a VHS and increasing its capacity for generating behaviour change, this seems unlikely given that the implementation intentions promoted by a VHS need to be encoded to memory to be later activated from memory to guide behaviour (e.g., Webb & Sheeran, 2004). A possible reason for the null results in this study is that the participants' lecture attendance rates were approaching ceiling. The observed attendance rate of 76% in the VHS only condition was not only 13 percentage points higher than the observed attendance rate in the control condition, it was substantially higher than the attendance rates that have typically been found in previous studies, which have been around 50% (e.g., Beovich et al., 2021; Hollett et al., 2020; Skead et al., 2020; Williams, 2022). It is possible, therefore, that there was limited scope to improve lecture attendance rates further with the encoding facilitation intervention.

It is also possible that the present sample had sufficient memory ability to enable the successful encoding and subsequent deployment of their specified implementation intentions, without the need for an encoding facilitation intervention. For each condition, the mean for the objective measure of memory ability was high. In all conditions, the participants achieved, on average, over 80% accuracy in the memory test. Although the participants' self‐reports indicated moderate levels of memory ability (means in the middle of the PRMQ scale), the standard deviations were small, indicating little variation around those moderate levels of memory ability. As a result, the scope within the present sample to find an added benefit of an encoding facilitation intervention, over and above a VHS, and the scope to detect differences in the efficacy of a VHS as a result of variations in memory ability, may have been limited. Further research might usefully test whether encoding facilitation interventions can boost the efficacy of VHSs, and whether memory ability can moderate the efficacy of VHSs, using samples with greater variation in memory ability. Given that memory difficulties tend to increase with age (e.g., Čepukaitytė et al., 2023; Lima‐Silva & Yassuda, 2009) samples with wider age ranges than typically found in general samples of university students could be employed. Further research might also usefully test the extent to which other mental strategies constitute effective interventions for boosting the efficacy of VHSs on lecture attendance rates. In other domains, mental contrasting (Oettingen et al., 2001) has been found to boost the efficacy of implementation intention interventions such as VHSs (e.g., Adriaanse et al., 2010; Kirk et al., 2013). Mental contrasting involves visualizing both a desired future outcome (e.g., lecture attendance) and the current obstacles or challenges (e.g., critical situations) that might stand in the way of achieving that outcome. It generates increases in motivation (e.g., goal intentions to attend lectures) and helps individuals identify which critical situations (e.g., in a VHS) need linking with goal‐directed responses to ensure a behaviour is carried out. Combining interventions that promote mental contrasting with VHSs might constitute a useful avenue for further research in the context of university students' lecture attendance.

### Possible limitations and considerations

A potential limitation of the current research is that the data were collected from a single university. It should be noted, however, that the proportion of First or Upper Second Class degree classifications that are awarded annually by the institution from which the present sample was drawn is comparable (within 10 percentage points higher or lower) with the proportion of these top degree classifications that are awarded by most (almost two‐thirds) of the other higher education institutions in the UK (Higher Education Statistics Agency, 2025). Given that academic achievement correlates with lecture attendance (e.g., Credé et al., 2010; Tokumitsu, 2017), the present findings are therefore likely to generalize widely. Nevertheless, it may be worthwhile for future research to test the efficacy of VHSs across the full range of academic institutions in terms of student achievement.

It should also be taken into account that females made up just over 80% of the sample. However, over 80% of students taking Psychology in UK higher education institutions are female (e.g., Johnson et al., 2020) and the present sample reflected the population in its demographic composition. That said, it might be useful for further research to investigate other cohorts containing predominantly male students, such as those found in Engineering subjects, where the proportion of males is over 80% (e.g., Engineering, 2023).

### Practical implications

Following Elliott et al. (2024), the findings from this study imply that VHSs could be used to help university students' increase their lecture attendance rates and achieve the associated academic benefits (e.g., Tokumitsu, 2017). VHSs do not suffer from many of the barriers that other behaviour change interventions do, such as limited resources for implementation, users' lack of understanding about content and effectiveness, or complicated implementation processes (e.g., Fu et al., 2023; Rogers et al., 2021). This is because they are self‐completed and, therefore, cost‐effective, supported by empirical evidence, and easy to administer. In the present context, they could be administered to university students at the beginning of each semester in paper and pencil format during introductory lectures. Alternatively, they could be administered electronically, as in the present study, through virtual learning environments (e.g., Learn VLE, Moodle) or smartphone applications, which are employed at several universities to help students manage their timetables. Incorporating VHSs into such applications would enable students to update and amend their implementation intentions, if required (e.g., to cope with new critical situations that might not have been anticipated at the start of semester).

## CONCLUSIONS

This research showed that a VHS increased the proportion of on‐campus lectures that university students attended over the course of an 11‐week teaching semester. There was no evidence that the efficacy of the VHS could be improved through mental rehearsal or that the intervention's effects on behaviour were moderated by memory ability. Further research testing the interplay between memory ability and implementation intention interventions (e.g., VHSs) could be usefully carried out in other domains where samples are likely to exhibit greater variance in memory ability. Further research in the context of university student behaviour could usefully test the extent to which other forms of intervention (e.g., mental contrasting or visualization) could boost the efficacy of VHSs.

## AUTHOR CONTRIBUTIONS

**Mark A. Elliott:** Conceptualization; investigation; funding acquisition; writing – original draft; writing – review and editing; visualization; methodology; validation; project administration; formal analysis; software; data curation; supervision; resources. **Allan McGroarty:** Conceptualization; investigation; funding acquisition; writing – original draft; writing – review and editing; visualization; validation; methodology; software; formal analysis; project administration; resources; supervision; data curation. **David J. Robertson:** Conceptualization; investigation; funding acquisition; writing – original draft; writing – review and editing; visualization; validation; methodology; software; formal analysis; project administration; resources; supervision; data curation. **Hazel P. Anderson:** Conceptualization; investigation; funding acquisition; writing – original draft; writing – review and editing; visualization; validation; methodology; software; formal analysis; project administration; resources; supervision; data curation.

## CONFLICT OF INTEREST STATEMENT

The authors have no conflicts of interest.

## Data Availability

The data that support the findings of this study are available from the corresponding author upon reasonable request.

## Footnotes

^1^
The achieved sample of *N* = 252 differed from the target sample of *N* = 432 that was specified in the pre‐registration for this project on the Open Science Framework. However, the target sample of *N* = 432 was based on an a priori power analysis that used an effect size estimate of *f* = 0.15, at α = 0.05 and power = 0.80 (Cohen, 1992). This was an ultra‐conservative estimate of effect size, smaller than the one found in Elliott et al.'s (2024) previous study on lecture attendance (*f* = 0.64). The achieved sample of *N* = 252 was still sufficient for detecting an effect size of *f* = 0.20 at α = 0.05 and power = 0.80. It was, therefore, still sufficient for detecting a meaningful sized effect, of the magnitude found in previous research. Given that the population was also exhausted, meaning there was no scope to collect more data, the data collection was terminated at *N* = 252.

^2^
The data were collected across two academic years for logistical reasons. There were no programme changes across the two academic years and attendance rates between semesters 1 and 2 are comparable, historically.

^3^
This “changing‐QR code” feature was disabled for students at level 1 of the curriculum due to technological problems. However, this was substituted with passwords, provided in lectures, which students needed to enter after scanning the QR codes in order to confirm their lecture attendances.
